# Within-Species Genomic Variation and Variable Patterns of Recombination in the Tetracycline Producer *Streptomyces rimosus*

**DOI:** 10.3389/fmicb.2019.00552

**Published:** 2019-03-21

**Authors:** Cooper J. Park, Cheryl P. Andam

**Affiliations:** Department of Molecular, Cellular, and Biomedical Sciences, University of New Hampshire, Durham, NH, United States

**Keywords:** *Streptomyces*, tetracycline, pan-genome, core genome, accessory genome, recombination, biosynthetic gene cluster

## Abstract

*Streptomyces rimosus* is best known as the primary source of the tetracycline class of antibiotics, most notably oxytetracycline, which have been widely used against many gram-positive and gram-negative pathogens and protozoan parasites. However, despite the medical and agricultural importance of *S. rimosus*, little is known of its evolutionary history and genome dynamics. In this study, we aim to elucidate the pan-genome characteristics and phylogenetic relationships of 32 *S. rimosus* genomes. The *S. rimosus* pan-genome contains more than 22,000 orthologous gene clusters, and approximately 8.8% of these genes constitutes the core genome. A large part of the accessory genome is composed of 9,646 strain-specific genes. *S. rimosus* exhibits an open pan-genome (decay parameter α = 0.83) and high gene diversity between strains (genomic fluidity φ = 0.12). We also observed strain-level variation in the distribution and abundance of biosynthetic gene clusters (BGCs) and that each individual *S. rimosus* genome has a unique repertoire of BGCs. Lastly, we observed variation in recombination, with some strains donating or receiving DNA more often than others, strains that tend to frequently recombine with specific partners, genes that often experience recombination more than others, and variable sizes of recombined DNA sequences. We conclude that the high levels of inter-strain genomic variation in *S. rimosus* is partly explained by differences in recombination among strains. These results have important implications on current efforts for natural drug discovery, the ecological role of strain-level variation in microbial populations, and addressing the fundamental question of why microbes have pan-genomes.

## Introduction

The gram-positive genus *Streptomyces* (phylum Actinobacteria) constitutes a highly diverse group that is widely distributed in nature. *Streptomyces* are prolific producers of bioactive specialized metabolites that have adaptive functions in nature and have found extensive utility in human medicine (Kinashi, 2011; Cruz-Morales et al., 2016; Xu et al., 2016). They are known as the major source of naturally derived antibiotics and many pharmaceutically relevant compounds (e.g., antifungals, antitumor, antihelmintics, antiprotozoans, immunosuppressants) (Kinashi, 2011). Many invertebrates such as wasps and ants also use the antibiotics produced by their *Streptomyces* symbionts to protect themselves against infection (Kaltenpoth et al., 2005; Seipke et al., 2012). In contrast to most bacteria, *Streptomyces* species are characterized by complex secondary metabolism and a fungal-like morphological differentiation that involves the formation of branching, filamentous vegetative growth and aerial hyphae bearing long chains of reproductive spores (Flärdh and Buttner, 2009); hence they were originally misclassified as fungi. The formation of aerial mycelium corresponds to the production of secondary metabolites such as antibiotics (Barka et al., 2016). Current estimate of the number of known *Streptomyces* species is approximately 650 (Labeda et al., 2012), making it one of the largest genera in the bacterial domain.

Whole genome sequencing of closely related, locally co-occurring microbial strains has revealed the existence of tremendous diversity within a species, arising from both allelic and gene content differences (Croucher et al., 2014; Zhu et al., 2015; Levade et al., 2017; Chang et al., 2018). Hence, using traditional taxonomic methods, it is difficult to delineate two lineages that are considered the same species yet vary substantially in gene content (Jaspers and Overmann, 2004; Segerman, 2012; Land et al., 2015). For example, fuzzy species i.e., those that that do not form clear, distinct species boundaries due to frequent gene exchange through recombination, have been reported in *Neisseria meningitidis* (Hanage et al., 2005). Hybrid lineages as in the case of *Klebsiella pneumoniae* sequence type [ST] 258 have been formed via a large chromosomal replacement event (Chen et al., 2014). Such genomic mosaicism and within-species variation can significantly impact a species’ response to selective pressures from antibiotic use, vaccination, immune responses and host environment (Brüggemann et al., 2018; Leventhal et al., 2018; Sela et al., 2018). Within-species genomic variation has also been reported to impact species divergence (Papke et al., 2007; Youngblut et al., 2015), metabolic diversity and versatility (Silby et al., 2011), and symbiotic relationships (De Maayer et al., 2014) in microbes, with medically relevant implications. For example, hyper-recombinant strains of *Streptococcus pneumoniae* are associated with the highest levels of drug resistance (Hanage et al., 2009). One important process that generates genomic variation in microbial species is recombination, the exchange of very similar DNA sequences between strains, and which can result to either the addition or replacement of homologous genes (Didelot and Maiden, 2010; Didelot et al., 2012). Most studies dealing with within-species genomic variation has been focused on antibiotic resistant pathogens [for example, (Grad et al., 2014; Andam et al., 2017; Grinberg et al., 2017; Lam et al., 2018)], yet rarely do we find investigations on antibiotic producers. In *Streptomyces*, genomic diversity between species has been widely investigated (Doroghazi and Metcalf, 2013; Kim et al., 2015; Andam et al., 2016; Huguet-Tapia et al., 2016), but the extent, origins and functional role of genomic variation among closely related strains of the same species remains poorly understood.

In this study, we focus on *Streptomyces rimosus*, which is best known as the primary source of the tetracycline class of antibiotics, most notably oxytetracycline (Petković et al., 2006). Tetracyclines are noted for their broad spectrum antibacterial activity and since the 1940s, have been used against a wide range of both gram-positive and gram-negative pathogens, mycoplasmas, chlamydiae, rickettsiae and protozoan parasites (Chopra and Roberts, 2001). Oxytetracycline, a well-studied polyketide natural product, is a bacteriostatic antibiotic that inhibits bacterial growth by reversibly binding to the 30S ribosomal subunit, thus inhibiting protein synthesis (Schnappinger and Hillen, 1996; Petković et al., 2006). *S. rimosus* is also known to produce the polyene antifungal rimocidin (Davisson et al., 1951). Although the precise mechanism of action of rimocidins is still not well understood, antifungal activity seems to be due to polyene molecules causing the sterol-containing cell membrane to become permeable (Seco et al., 2005). Despite the medical and agricultural importance of *S. rimosus* and the variety of antibiotics it produces, little is known of its evolutionary history and genome characteristics. Here, we explore the pan-genome characteristics and phylogenetic relationships of 32 *S. rimosus* genomes. We report high levels of inter-strain genomic variation, including the differential distribution and abundance of BGCs among strains. BGCs represent a collection of genes that, together are responsible for the production of a specific secondary metabolite, such as antibiotics. We also observed high frequency of recombination which may partly explain the large genomic variation among strains; however, recombination is biased, with some strains exhibiting more frequent donation or receipt of DNA than other strains. These results have important implications on current efforts for natural drug discovery, the ecological role of strain-level genomic variation in microbial populations, and addressing the fundamental question of why microbes have pan-genomes.

## Materials and Methods

### Dataset

A total of 32 genomes of *S. rimosus* available in November 2018 were downloaded from the RefSeq database of the National Center for Biotechnology Information (NCBI). Accession numbers and genomic information (genome size, % GC content, number of genes, number of protein-coding genes) are shown in Supplementary Table S1. To maintain consistency in gene annotations, the genomes were re-annotated using Prokka with default parameters (Seemann, 2014).

### Pan-Genome and Phylogenetic Analysis

Core and accessory genes were identified using Roary with default settings (Page et al., 2015). Roary iteratively pre-clusters protein sequences using CD-HIT (Fu et al., 2012), a fast program for clustering and comparing, which results to a substantially reduced set of data. Sequences in this reduced dataset were compared using all-against-all BLASTP (Altschul et al., 1990) and were then clustered the second time using Markov clustering (Enright et al., 2002). Each orthologous gene family from the merged CD-HIT and MCL were aligned using MAFFT (Katoh et al., 2002). We used Phandango (Hadfield et al., 2018) to visualize the presence-absence of genes per strain. The gene sequence alignments of each identified core gene family were concatenated to give a single core alignment, and a maximum-likelihood phylogeny was then generated using the program RAxML v.8.2.11 (Stamatakis, 2006)with a general time reversible (GTR) nucleotide substitution model (Tavaré, 1986), four gamma categories for rate heterogeneity and 100 bootstrap replicates. The phylogenetic tree was visualized using the Interactive Tree of Life (iToL) (Letunic and Bork, 2016).

We used the program micropan (Snipen and Liland, 2015) implemented in R (R Core Team, 2013) to calculate the pan-genome’s decay parameter (α) (Tettelin et al., 2008) and genomic fluidity (φ) (Kislyuk et al., 2011). The decay parameter measures the number of new gene clusters observed when genomes are ordered in a random way, which provides an indication of the openness or closeness of a pan-genome (Tettelin et al., 2008). An open pan-genome indicates that the number of new genes to be observed in future genomes is large, while a closed pan-genome indicates that after a certain number of sequenced genomes are added, the number of new genes discovered reaches a plateau (Tettelin et al., 2008). The genomic fluidity is a measure of the dissimilarity of genomes based on the degree of overlap in gene content and is defined as the number of unique gene families divided by the total number of gene families (Kislyuk et al., 2011). Both metrics are used to evaluate within-species genomic variation. Genome-wide average nucleotide identity (ANI) of all orthologous genes shared between any two genomes was calculated for all possible pairs of genomes (Jain et al., 2018). ANI is a robust similarity metric that has been widely used to resolve inter- and intra-strain relatedness. The threshold value of 95% has been widely used as a cutoff for comparisons belonging to the same or different species (Jain et al., 2018).

Biosynthetic gene clusters encoding secondary metabolites were predicted and annotated using the standalone version of antiSMASH 4.1 (Blin et al., 2017). antiSMASH predicts BGCs using signature profile Hidden Markov Models (pHMMs) derived from multiple sequence alignments of experimentally characterized signature proteins or protein domains of known BGCs (Blin et al., 2017). It then aligns the identified regions at the gene cluster level to their nearest relatives from a database containing all other known gene clusters (Blin et al., 2017). BGCs that encode for oxytetracycline and rimocidin were identified by searching all the genomes for homologs of each of the genes comprising the two BGCs using BLASTP (Altschul et al., 1990) with a minimum *e*-value of 10^-10^. Individual genes in a BGC obtained from previous studies (Seco et al., 2005; Zhang et al., 2006) were used as query sequences. Presence of the BGC was ascertained if there were BLASTP hits for at least 90% of the genes within the BGC. Sequences for the individual genes of the two BGCs were obtained from the Database of BIoSynthesis cluster CUrated and InTegrated (DoBISCUIT) (Ichikawa et al., 2013) based on previous studies of the oxytetracycline and rimocidin BGCs (Seco et al., 2005; Zhang et al., 2006).

### Recombination Detection

We used three approaches to detect recombination in the population. First, the pairwise homoplasy index or PHI (Φ*w*) test was used to determine the statistical likelihood of recombination being present in our dataset (Bruen et al., 2006). This statistic measures the genealogical correlation or similarity of adjacent sites. Under the null hypothesis of no recombination, the genealogical correlation of adjacent sites is invariant to permutations of the sites as all sites have the same history (Bruen et al., 2006). Significance of the observed Φ*w* was obtained using a permutation test. We then visualized potential recombination events using Splitstree v.4.14.4, which integrates reticulations due to recombinations in phylogenetic relationships rather than forcing the data to be represented in a bifurcating tree (Huson and Bryant, 2006). Next, we ran fastGEAR (Mostowy et al., 2017) with default parameters to detect genome-wide mosaicism. Using the individual sequence alignments of all core and shared accessory genes, sequence clusters were first identified using BAPS (Cheng et al., 2013) implemented in fastGEAR. fastGEAR infers the population structure of individual alignments using a Hidden Markov Model to identify lineages in an alignment. Lineages are defined as groups which are genetically distinct in at least 50% of the alignment. Within each lineage, recombinations are identified by comparing every nucleotide site in the target sequence to all remaining lineages and asks whether it is more similar to something else compared to other strains in the same lineage. In other words, fastGEAR infers recombination by searching for similar nucleotide segments between diverse sequence clusters. To test the significance of the inferred recombinations and identify false-positive recombinations, fastGEAR uses a diversity test, wherein the diversity of the fragment in question is different compared to its background. To predict the origin of the recently recombined regions, the sequences on which the recombination event was predicted to have occurred were first extracted from the genome data. The recombined regions were then used as query sequences in BLASTN (Altschul et al., 1990) searches against all possible genomes from the identified donor lineage as well as from the non-redundant (nr) nucleotide database in NCBI. The top BLAST hit with the highest bit score was considered as the potential donor, provided that the hit covered at least 50% of the recombination fragment length and had a minimum of 99% nucleotide identity.

## Results

### Pan-Genome Characteristics of *S. rimosus*

**Figure 1:**
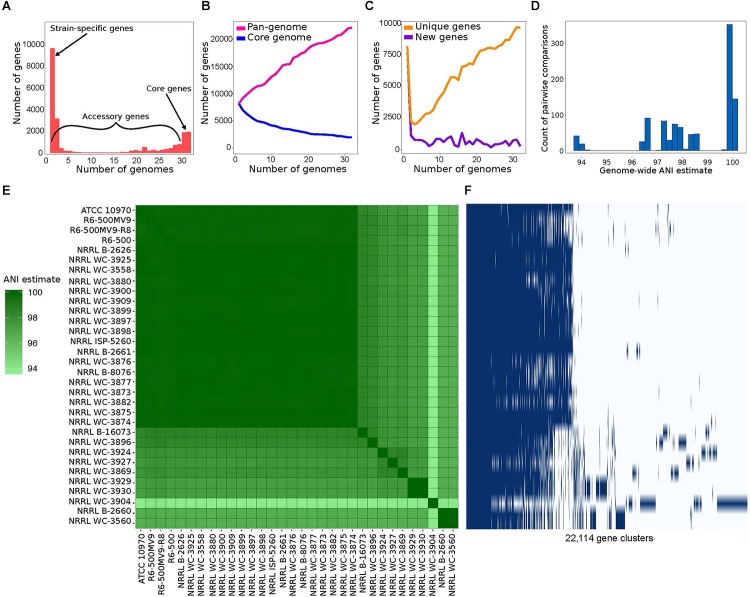
Pan-genome analysis of 32 *Streptomyces rimosus* strains. **(A)** The number of unique genes that are shared by any given number of genomes or unique to a single genome. Numerical values for each gene category are shown in Supplementary Table S2. **(B)** The size of the core genome, i.e., genes that are present in at least 31 of the 32 strains (blue line) and pan-genome, i.e., the totality of unique genes present in the population (pink line) in relation to numbers of genomes compared. The list of core genes is listed in Supplementary Table S3. **(C)** The number of unique genes, i.e., genes unique to individual strains (yellow line) and new genes, i.e., genes not found in the previously compared genomes (purple line) in relation to numbers of genomes compared. **(D)** Distribution of pairwise average nucleotide identity (ANI) values. ANI calculates the average nucleotide identity of all orthologous genes shared between any two genomes. The 95% ANI cutoff is a frequently used standard for species demarcation. **(E)** Pairwise whole genome ANI comparison. Percentage values are shown in Supplementary Table S4. **(F)** Gene presence-absence matrix showing the distribution of genes present in a genome. Each row corresponds to a strain in panel **E**. Each column represents an orthologous gene family. Dark blue blocks represent the presence of a gene, while light blue blocks represent the absence of a gene.

We used a total of 32 *S. rimosus* genomes downloaded from the RefSeq database of NCBI (Supplementary Table S1). Genome sizes range from 8.14 to 10.02 Mb (mean = 9.20 Mb), while the number of predicted genes per genome ranges from 7,071 to 8,666 (mean = 8,020). The % G+C content also varies among genomes, ranging from 71.7 to 72.1%. We used Roary (Page et al., 2015) to calculate the *S. rimosus* pan-genome, defined as the totality of genes present in a group of genomes (Page et al., 2015). Roary classifies orthologous gene families into core genes and accessory genes. Core genes are present in 99% ≤ strains ≤ 100% (Supplementary Tables S2, S3). To take sequencing and assembly errors into account, Roary also calculates the number of soft core genes which are present in 95% ≤ strains < 99%. Accessory genes comprise the shell genes which are present in 15% ≤ strains < 95% and cloud genes which are present in < 15% of strains (Figure 1A). We found a considerably small core genome (1,945 genes) comprising 8.8% of the pan-genome (22,114 genes). Broadening our definition of the core genome to incorporate the soft core genes still only represented approximately 17% of the total pan-genome. The core genome comprises 22.44–27.51% of each individual genome. It is also notable that the vast majority of accessory genes (9,646, representing 44% of the pan-genome) are unique to a single strain. In microbes, large accessory genomes and high number of strain-specific genes are often associated with horizontal gene transfer (HGT) (Pohl et al., 2014; Vos et al., 2015; Zhu et al., 2016).

The size of the pan-genome and its increase/decrease in size upon addition of new strains can be used to predict the future rate of discovery of novel genes in a species (Medini et al., 2005; Tettelin et al., 2008). We used the program micropan to estimate the openness of the *S. rimosus* pan-genome by using the Heap’s power law function (Tettelin et al., 2008) for all possible permutations of all *S. rimosus* genomes. We calculated the decay parameter α, wherein an α > 1.0 indicates that the size of the pan-genome approaches a constsant as more genomes are sampled (i.e., the pan-genome is closed), while α < 1.0 indicates that the size of the pan-genome is increasing and unbounded by the number of genomes considered (i.e., the size of the pan-genome follows Heaps’ law and the pan-genome is open) (Medini et al., 2005; Tettelin et al., 2008). We obtained an α = 0.83 using 100 permutations in *S. rimosus* and suggests an open pan-genome; hence, we are likely to find new genes as more genomes are sequenced in the future. The openness of a pan-genome reflects the diversity of the gene pool within bacterial species, and is often associated with bacterial species that inhabit multiple environments or have different mechanisms and opportunities for gene exchange (Rouli et al., 2015; Brito et al., 2018). We find that the pan-genome of *S. rimosus* increases with the addition of new genomes, while the core genome decreases and begins to plateau at approximately 20 genomes (Figure 1B). The number of new, previously unseen, genes found as each genome is added to the plot averages 450 (Figure 1C). Finally, we also show the number of unique genes overall that have been observed exactly once continues to increase as each genome is added (Figure 1C).

To estimate the degree of overlap with respect to gene cluster content between any two genomes, we also calculated the genomic fluidity (φ), which provides an overview of gene-level similarity between genomes and is defined as the number of unique gene families divided by the total number of gene families (Kislyuk et al., 2011). Fluidity values range from 0 to 1, with 0.0 to indicate that the two genomes contain identical gene clusters, while 1.0 if the two genomes are non-overlapping (Kislyuk et al., 2011). Hence, a fluidity value of 0.2 for example implies that 20% of the genes are unique to their host genome and the remaining 80% are shared between genomes (Halachev et al., 2011). We obtained a genomic fluidity value of 0.12, which suggests that *S. rimosus* has a high degree of genomic diversity and is within the range found in other bacterial species (Halachev et al., 2011; Kislyuk et al., 2011).

To determine the degree of genomic relatedness and hence clarify whether these 32 genomes belong to the same species, we calculated the pairwise ANI for all possible pairs of genomes. ANI calculates the average nucleotide identity of all orthologous genes shared between any two genomes and organisms belonging to the same species typically exhibit ≥95% ANI (Jain et al., 2018). The distribution of pairwise ANI values reveal that the *S. rimosus* genomes are within the 95% cutoff and should therefore considered the same species (Figure 1D,E and Supplementary Table S4). Strain NRRL WC-3904 exhibits a slightly lower ANI value of 94% when compared to the rest of the genomes in the dataset. To further visualize the distribution of genes among the strains, we generated a pan-genome matrix using Roary and Phandango (Figure 1F). We find that NRRL WC-3904 exhibits a highly divergent accessory genome profile compared to the remaining 31 genomes, which may explain its slightly lower ANI values.

### Strain-Level Variation in the Distribution and Abundance of BGCs

*Streptomyces* are renowned for their ability to produce structurally diverse natural products (called secondary metabolites), many of which are widely used in medicine, agriculture and bioenergy processes. Secondary metabolites differ from primary metabolites in that they are not involved in essential metabolic activities required for normal growth and reproduction of the organism, but may contribute significantly to an individual’s fitness and ecological adaptation (Zotchev, 2014). Mining bacterial genomes has shown that their potential for producing secondary metabolites and other bioactive compounds is much higher than what is observed in the laboratory (Doroghazi and Metcalf, 2013), and hence has important implications in discovering novel bioactive compounds.

**Figure 2:**
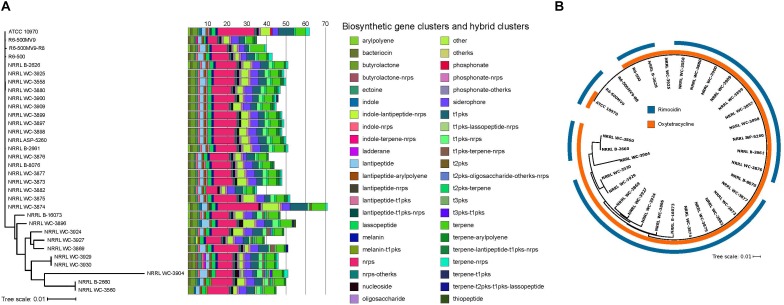
Distribution of BGCs per genome. **(A)** BGCs and hybrid clusters were identified using antiSMASH. The maximum likelihood phylogenetic tree was reconstructed using concatenated alignments of 1,945 core genes. Scale bar of phylogenetic tree represents nucleotide substitutions per site. Acronyms: nrps, non-ribosomal peptide synthase; t1pks, type 1 polyketide synthase; t2pks, type II polyketide synthase; t3pks, type III polyketide synthase; ks, ketosynthase. **(B)** Phylogenetic distribution of the oxytetracycline and rimocidin BGCs. Colored rings outside the tree show the presence/absence of BGCs known to encode for oxytetracycline and rimocidin. The two BGCs were identified by searching all the genomes for homologs of each of the genes comprising the BGCs using BLASTP (Altschul et al., 1990) with a minimum *e*-value of 10^-10^. Individual genes in a BGC obtained from previous studies (Seco et al., 2004; Zhang et al., 2006) were used as query sequences. Presence of the BGC was inferred if there were significant BLASTP hits for at least 90% of the individual genes within the BGC.

Biosynthesis of secondary metabolites is typically governed by 10–30 genes organized as clusters in the genome, allowing the coordinated expression of the genes involved in their biosynthesis, resistance and efflux (Zotchev, 2014). We used antiSMASH 4.1 (Blin et al., 2017) to identify BGCs present in each *S. rimosus* genome. Each genome harbors 35–71 BGCs, with more than half of the BGCs predicted to produce polyketide (PKS) and non-ribosomal peptide synthetase (NRPS), or hybrids of the two (Figure 2A). This range in BGC content in *S. rimosus* is consistent with results from previous BGC surveys in other *Streptomyces* species (Seipke et al., 2011; Seipke et al., 2015; Choudoir et al., 2018; Vicente et al., 2018) and the widely studied actinobacterium *Salinispora* (Udwary et al., 2007; Letzel et al., 2017), although many BGCs often remain “silent” under standard laboratory conditions (Bentley et al., 2002; Ikeda et al., 2003; Ohnishi et al., 2008). Hybrid BGCs contain genes that code for more than one type of scaffold-synthesizing enzymes (Cimermancic et al., 2014; Zotchev, 2014). Many of the NRPS or PKS hybrids are found in one or few genomes: lantipeptide-t1pks-nrps hybrid in two genomes, melanin-t1pks hybrid in two genomes, phosphonate in one genome, t1pks-lassopeptide-nrps hybrid in two genomes, terpene-t2pks-t1pks-lassopeptide hybrid in two genomes, and terpene-t2pks-t1pks-lassopeptide hybrid in one genome. Aside from NRPS and PKS, other commonly shared BGCs are bacteriocin, butyrolactone, ectoine, lantipeptide, lasso peptide, melanin, nucleoside, siderophore, and terpene. Other BGCs are also differentially distributed among the 32 genomes: indoles in five genomes, ladderane in two genomes, phosphonate in one genome, and thiopeptide in one genome. Interestingly, Type II PKS and its hybrids were detected in 29 strains. Type II PKS synthesize tetracyclines and other aromatic polyketides such as anthracyclines, angucyclines, and pentangular polyphenols, which are also widely used as antibiotics or chemotherapeutics (Hertweck et al., 2007; Kim and Yi, 2012). Overall, we find that each individual *S. rimosus* genome harbors a unique combination of BGCs, further highlighting the extent of inter-strain genomic variation in *S. rimosus*. We note, however, that the reported numbers have likely been overestimated due to the low quality of some of the genome assemblies, which can affect the accurate BGC prediction in antiSMASH.

*Streptomyces rimosus* is particularly well known for its production of the antibiotics oxytetracycline and rimocidin (Petković et al., 2006). To determine the presence of BGCs that encode for these two antibiotics, we used BLASTP to search the 32 genomes for the individual genes of each BGC (Figure 2B and Supplementary Tables S5, S6). We found that, except for a single genome (R6-500MV9-R8), all *S. rimosus* genomes carry one or both BGCs. A total of 30 genomes had nearly 100% matches for each of the 21 genes found in the oxytetracycline BGC, while two showed a match for only a single gene. On the other hand, the rimocidin BGC was detected in 28 genomes (Supplementary Tables S7, S8).

### Frequent but Biased Recombination Between Strains

**Figure 3:**
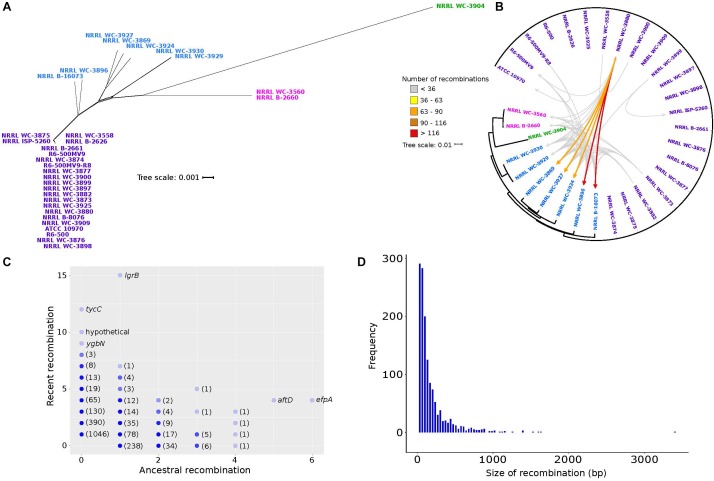
Genetic relationships among *S. rimosus* strains are influenced by homologous recombination. **(A)** A phylogenetic network of the *S. rimosus* core genome generated using SplitsTree. The strain names were colored according to clustering results using BAPS. **(B)** Donor-recipient linkages of major recombination events (i.e., highways of recombination) identified using fastGEAR and BLASTN. Scale bar represents nucleotide substitutions per site. Each arrow represents a certain number of recombination events between a pair of genomes, with different colors representing the range of numbers. **(C)** Genes that have undergone recent or ancestral recombination. Horizontal axis shows the estimated number of ancestral recombinations and vertical axis shows the estimated number of recent recombinations. Names of some of the genes are shown. Numbers in parenthesis indicate the number of genes represented by overlapping dots found on the same position. **(D)** Frequency histogram of the size of recombination events of all genes in the pan-genome.

In *Streptomyces*, recombination is known to have greatly contributed to shaping its evolution and diversity, with some taxonomically recognized species exhibiting significant genetic mosaicism (Doroghazi and Buckley, 2010; Andam et al., 2016; Cheng et al., 2016). To infer the phylogenetic relationships of the 32 *S. rimosus* genomes, the 1,945 core genes were aligned and concatenated, giving a total length of 2,017,766 bp. The core genome phylogeny reveals four clusters (Figure 3). Under the null hypothesis of no recombination, we calculated the PHI statistic (Bruen et al., 2006) and detected evidence for significant recombination in the core genome (*p*-value = 0.0). Recombination in *S. rimosus* core genome can be visualized using Neighbor Net implemented in SplitsTree4, which shows the reticulations in their phylogenetic relationships (Huson, 1998; Figure 3A). To further characterize the extent of genome-wide recombination in *S. rimosus*, we ran fastGEAR (Mostowy et al., 2017) on individual sequence alignments of core and shared accessory genes. Each predicted recombination fragment was then used as a query in BLASTN (Altschul et al., 1990) against the predicted lineage of donors to identify the most likely donor-recipient linkages. We found that recombination is frequent, with a total of 2,148 genes that had experienced recombination. However, when we mapped the donor-recipient recombination partners, we found that although recombination is frequent, it does not impact all genomes similarly (Figure 3B). A total of 12 genomes were not identified to be either a donor or recipient of recombined DNA. Of those genomes wherein recombination was detected, there were genomes that appear to accept more recombined DNA than others. We calculated the number of recombination events for any genome pair that is at least one standard deviation above the group’s average of 36 recombination events. We identified five genomes (NRRL WC-3869, NRRL WC-3927, NRRL WC-3924, NRRL WC-3896, NRRL B-16073) that have received more recombined DNA than others. We observed that although recombination is frequent, it does not impact all 32 genomes similarly. We find that recombination is biased, with some strains receiving more recombined DNA more often (NRRL WC-3896 and B-16073), while others exhibit preferences to specific exchange partners (Figure 3B).

The strength of fastGEAR is its ability to identify both recent (affecting a few strains) and ancestral (affecting entire lineages) recombinations (Mostowy et al., 2017). Of the recent recombination events identified, we observed a total of 91 unique donor-recipient pairs and five of these pairs contributed 49% or more of the total recombination events (Figure 3B). A total of 30 recent recombination events originate from donors outside of the *S. rimosus* dataset. Of these, half came from other *Streptomyces* species and two from other genera in Actinobacteria (*Micromonospora* and *Rhodococcus*). The taxonomic origins of the remaining recombination events could not be precisely determined due to the short length of the recombined sequences. Finally, we found that, of the 22,114 genes that comprise the pan-genome, a total of 2,149 genes have had a history of recombination (Figure 3C). Of these, 1,147 genes were involved in recent recombination and 386 genes in ancestral recombination. The most frequently recombined genes include those associated with antibiotic biosynthesis (*lgrB*, *tycC*), transmembrane transport (*ygbN*, *efpA*) and transferase (*aftD*) (Figure 3C and Supplementary Table S9).

The lengths of the recombined regions have an approximately exponential distribution, with majority of recombination events being small (<500 bp) and large events occurring relatively infrequently (Figure 3D). The median length of recombined fragments is 230 bp and the largest recombination event is 11,934 bp in strain NRRL B-16073. Our finding of a heterogeneous model of recombination is consistent with those reported in other bacterial species, such as the pathogens *Streptococcus pneumoniae* (Chewapreecha et al., 2014) and *Legionella pneumophila* (David et al., 2017), and our results demonstrate that it also holds true for non-pathogenic species. The observed heterogeneity in recombination sizes has been previously described and classified into micro-recombinations (i.e., short, frequent sequence replacements) and macro-recombinations (i.e., rarer, multi-fragment, saltational sequence replacements) (Mostowy et al., 2014), and our results are consistent with these. Overall, our analysis of recombination in *S. rimosus* reveals inter-strain variation in terms of the frequency of DNA donation or receipt, genes that experience the most frequent recombination and the size of recombination events.

## Discussion

The tremendous diversity and ability of *Streptomyces* to inhabit numerous ecological niches and produce diverse clinically useful compounds have been attributed to their large pan-genomes (Zhou et al., 2012; Kim et al., 2015). In a recent study of 122 *Streptomyces* genomes comprising multiple species, a mere 2.63% (*n* = 1,048 genes present in ≥95% of all genomes) of the 39,893 gene families present constitutes the core genome while the remaining genes are classified as accessory genes (McDonald and Currie, 2017). At the species level, our results on 32 *S. rimosus* genomes reveal similar patterns of having a small fraction of core genes (*n* = 1,945 genes) which make up 8.8% of a much larger pan-genome (22,114 genes). When we include the soft core genes (genes present in at least 95% of the strains) numbering 1,874 genes, the core genome still represents only 17% of the pan-genome. While sequencing errors and the draft nature of the genomes used here may partly explain the low number of core genes in *S. rimosus*, the observation of a small core genome in microbial species is not uncommon and has been reported in other species (McInerney et al., 2017), including Actinobacteria. For example, the core genome of 28 *Bifidobacterium longum* subsp. *longum* strains consists of 1,160 genes from a pan-genome of 4,169 genes (Chaplin et al., 2015). In 18 strains of *Corynebacterium pseudotuberculosis*, the core genome consists of 1,355 genes and a pan-genome of 3,183 genes (Baraúna et al., 2017). In an analysis of 2,085 *Escherichia coli* genomes, the largest pan-genome analysis to date, a total of 3,188 genes comprises the core genome and is a remarkably small number compared to the stunning 90,000 genes that comprise the *E. coli* pan-genome, with a third of these genes occurring in only one genome (Land et al., 2015). The open pan-genome of *S. rimosus* means that the sequencing of new genomes will possibly add new genes not described in this current pan-genome study. Lastly, while it is difficult to speculate on the causes of why one strain (NRRL WC-3904) has an ANI of 94% compared to the other genomes (slightly below the 95% cutoff for species delineation), previous ANI-based studies have found similar results and may reflect the edge of a genetic discontinuum between species (Caro-Quintero et al., 2009; Jain et al., 2018). However, using the 83% ANI cutoff to delineate different species (Jain et al., 2018), WC-3904 cannot be classified as a separate species.

Compared to other Actinobacteria species and other bacterial phyla, *Streptomyces* also harbors the highest numbers of secondary metabolite BGCs from a large variety of classes and often with little overlap between strains (Doroghazi and Metcalf, 2013). Here, each *S. rimosus* genome harbors a unique repertoire of BGCs ranging from 35 to 71 BGCs per genome, including many NRPS, PKS and hybrid clusters. These results highlight the importance of sampling multiple strains of the same species in improving efforts for natural drug discovery. Antibiotics with new inhibitory mechanisms or cellular targets are urgently needed as resistance to our existing arsenal of drugs is growing and multidrug resistance becomes widespread. While emergence of resistance to and decreased effectiveness of existing tetracyclines as front-line antibiotics have grown over the years (Chopra and Roberts, 2001), our genomic analyses suggest that the potential of *S. rimosus* as producers of novel antibiotics has not been fully explored and many natural products are yet to be discovered from this species.

Only recently with whole genome sequencing do we come to recognize the extent in which, within each bacterial species, different strains may vary in the set of genes they encode (Konstantinidis et al., 2006; Seipke, 2015; Leonard et al., 2016; Truong et al., 2017). Recently, a polyphasic analyses was conducted on ten strains closely related to *Streptomyces cyaneofuscatus*, with all strains having identical 16S rRNA sequences (Antony-Babu et al., 2017). Authors reported significant differences in morphological, phenotypical and metabolic characteristics, and could in fact be distinguished as five different species (Antony-Babu et al., 2017). Such variation is not uncommon and has been reported to influence functions relevant to the structure and dynamics of the entire microbial community, adaptation to changes in the environment, and interactions with the eukaryotic host (Greenblum et al., 2015). However, the large pan-genome size of a microbial species remains intriguing. Efforts to elucidate the factors that shape and maintain the existence of a multitude of genes in a few strains have recently demonstrated the contributions of selection, drift, recombination, migration, and effective population size (Andreani et al., 2017; McInerney et al., 2017; Vos and Eyre-Walker, 2017; Bobay and Ochman, 2018). While the relative contributions of these processes across multiple microbial species remain unclear, it is likely that one or few of these processes may explain the large pan-genome size of *S. rimosus*.

Equally intriguing is our observation of heterogeneity in the frequency and characteristics of recombination. We observed that some strains donate or receive DNA more often than others, while some strains tend to frequently recombine with specific partners. Such a pair of strains or lineages exchanging DNA more often between them than with others is said to be linked by a highway of gene sharing (Beiko et al., 2005; Bansal et al., 2013). A highway of recombination between a pair of genomes, wherein they exchange DNA more often between them than with others, are likely to represent specific lineages that function as hubs of gene flow, facilitating the rapid spread of genes (for example, those associated with antibiotic resistance, metabolic genes, niche-specific genes) (Chewapreecha et al., 2014). These highways have been previously identified at higher taxonomic groups (domains, phyla, families) (Beiko et al., 2005; Zhaxybayeva et al., 2009; Bansal et al., 2013), but have only recently been reported at the sub-species level (Chewapreecha et al., 2014). However, the drivers of heterogeneity in the frequency and characteristics of recombination among members of the same species is poorly understood. Biases in recombination partners and other forms of genetic exchange have been reported to arise from phylogenetic relatedness (including compatible mismatch repair systems), geographical or physical proximity, shared ecological niches, or common set of mobile elements (Beiko et al., 2005; Andam and Gogarten, 2011; Smillie et al., 2011; Skippington and Ragan, 2012). However, it is unclear whether this variation in recombination is adaptive or not at the population level, to what extent strains that less often recombine benefit from the population, and how the population evolves with a mix of strains that vary in recombination frequencies and partners. In the future, a possible approach to further understand the variation in the recombination process in microbial genomes is to integrate evolutionary game theory with genome sequencing of closely related bacterial strains, composed of recombining (“cooperators”) and non-recombining (“cheaters”) that can be modeled over hundreds of generations (Van Dyken et al., 2013; Rauch et al., 2017; Zomorrodi and Segrè, 2017).

The principal caveat in this analysis is that that the quality of the *S. rimosus* genomes we examined are of varying quality, with some genomes having several hundred contigs. The draft nature of the genomes can have a significant impact on the antiSMASH output, particularly so in the identification of hybrid BGCs. There are two reasons for this. First, antiSMASH is conservative in terms of predicting the borders of BGCs and second, most strains harbor BGC islands on the arms of linear chromosomes [as in *Streptomyces* (Kinashi, 2011)], which antiSMASH can misidentify as hybrid BGCs. Another important limitation is that NCBI did not have information about the specific ecological and/or geographical origins of these strains (Supplementary Table S1). Moreover, only 32 genomes were considered. Because the size of the core and accessory genomes is a function of the number and characteristics of the dataset, improved sequencing quality as well as the sequencing of additional genomes is likely to alter some of our results. In our study, we found evidence of an open *S. rimosus* pan-genome (i.e., the number of new genes discovered increases with the number of additionally sequenced strains) even with the use of draft genomes. Hence, we may expect to find a larger core genome and additional accessory genes if these 32 strains are re-sequenced and complete genomes are generated. We also expect to find additional new and unique *S. rimosus* genes from strains inhabiting diverse environments. While they are most prevalent in soil and decaying vegetation, many *Streptomyces* species have also been identified in extreme environments and the gut of insects (Barka et al., 2016; van der Meij et al., 2017). In these places, we are likely to find niche-specific genes (Croucher et al., 2014; Gupta et al., 2015; Zhu et al., 2016), further expanding the size of the accessory genomes of *Streptomyces* species. Much of the work on *Streptomyces* isolation have only concentrated on soil environments, but future work should increase sampling efforts of *S. rimosus* in previously unexplored niches.

## Conclusion

In this study, we focus on elucidating the pan-genome characteristics and phylogenetic relationships of 32 *S. rimosus* genomes, which is best known as the primary source of the tetracyclines used against many species of pathogens and parasites. There are two major conclusions from this study. First, *S. rimosus* exhibits tremendous inter-strain genomic and biosynthetic variation, which suggests that their potential as an antibiotic producer remains to be fully explored. Second, we observed high levels of recombination between strains; however, recombination is not a homogenous process in this species. Our findings contribute to addressing the puzzle of why microbes have pan-genomes (Andreani et al., 2017; McInerney et al., 2017; Vos and Eyre-Walker, 2017; Bobay and Ochman, 2018) and the contributions of biased gene exchange to maintaining gene content variability within a species (Beiko et al., 2005; Andam and Gogarten, 2011; Bansal et al., 2013; Chewapreecha et al., 2014).

## Data Availability

Publicly available datasets were analyzed in this study. This data can be found here: https://www.ncbi.nlm.nih.gov/genome/?term=streptomyces%20rimosus.

## Author Contributions

CA and CP designed the work and wrote the manuscript. CP performed all bioinformatics analyses. CA guided the work. Both authors read and approved the final manuscript.

## Conflict of Interest Statement

The authors declare that the research was conducted in the absence of any commercial or financial relationships that could be construed as a potential conflict of interest.

## Supplementary Material

TABLE S1 Accession numbers and genome characteristics of *S. rimosus* genomes used in this study.

TABLE S2 Number of genes in each gene category of the pan-genome.

TABLE S3 List of core genes inferred by Roary.

TABLE S4 ANI values (%) for each pairwise genome comparison in Figure 1E.

TABLE S5 List of genes in the oxytetracycline BGC used in Figure 2B. Sequences were obtained from DoBISCUIT (Database of BIoSynthesis cluster CUrated and InTegrated) (Ichikawa et al., 2013).

TABLE S6 List of genes in the rimocidin BGC used in Figure 2B. Sequences were obtained from DoBISCUIT (Database of BIoSynthesis cluster CUrated and InTegrated) (Ichikawa et al., 2013).

TABLE S7 BLAST results for individual genes in the oxytetracycline BGCs. Numbers represent the percentage sequence similarity to the query sequence.

TABLE S8 BLAST results for individual genes in the rimocidin BGC. Numbers represent the percentage sequence similarity to the query sequence.

TABLE S9 List of recombined genes inferred by fastGEAR.
