# Supplementary material for: Within-Species Genomic Variation and Variable Patterns of Recombination in the Tetracycline Producer Streptomyces rimosus
Source: Front Microbiol. 2019 Mar 21;10:552. doi: 10.3389/fmicb.2019.00552 (PMC6437091; doi:10.3389/fmicb.2019.00552)
Supplement: TABLE S2 — Number of genes in each gene category of the pan-genome. [file Table_2.DOCX]

Supplementary Table S2. Number of genes in each gene category of the pan-genome

| Gene category | Number of genes |
| --- | --- |
| Core genes       (99% <= strains <= 100%)  Soft core genes (95% <= strains < 99%)  Shell genes     (15% <= strains < 95%)  Cloud genes     (0% <= strains < 15%)  Total genes     (0% <= strains <= 100%) | 1,945  1,874  4,898  13,397  22,114 |
